# Aging-related severe hypertension causes prostatic gland atrophy and testicular injury in rats

**DOI:** 10.1038/s41598-026-41624-x

**Published:** 2026-03-03

**Authors:** Shogo Shimizu, Yoshiki Nagao, Atsushi Kurabayashi, Yoshihiro Murata, Mutsuo Taniguchi, Masahiro Yamaguchi

**Affiliations:** 1 Department of Physiology, Kochi Medical School, Kochi University, Nankoku, 783-8505 Japan; 2 Department of Pediatrics, Kochi Medical School, Kochi University, Nankoku, Japan; 3 Department of Pathology, Kochi Medical School, Kochi University, Nankoku, Japan

**Keywords:** Aging, Hypertension, Prostatic atrophy, Prostatic hyperplasia, Testis, Diseases, Medical research, Physiology, Urology

## Abstract

The combined effects of aging and hypertension on the male reproduction system remain unclear. We investigated these effects on prostate and testis from histological and endocrinological perspectives. Male spontaneously hypertensive rats (SHRs) and normotensive Wistar Kyoto rats (WKYs) (n = 8 per group) were evaluated at 36 weeks (adult) and 72 weeks (aged). Compared with age-matched WKYs, adult SHRs showed significantly higher prostate weight, prostate weight-to-body weight ratio and glandular epithelial area in the ventral prostate. On the contrary, aged SHRs showed significantly lower prostate weight and outer glandular perimeter than age-matched WKYs. At both ages, SHRs had significantly higher blood pressure and lower prostatic blood flow than age-matched WKYs, and aging-dependent increase in the blood pressure was observed in SHRs but not in WKYs. Aged SHRs had significantly lower serum testosterone levels, higher testicular weight-to-body weight ratios, seminiferous epithelial desquamation and neutrophil infiltration than aged WKYs, while these alterations were not obvious in adult SHRs. In conclusion, whereas adult SHRs exhibit prostatic hyperplasia, aged SHRs exhibit severe hypertension, prostatic atrophy, testicular injury, and reduced serum testosterone levels. Aging-related severe hypertension may be a risk factor for prostatic atrophy which might be related to reduced testosterone levels.

**Supplementary Information:**

The online version contains supplementary material available at 10.1038/s41598-026-41624-x.

## Introduction

Benign prostatic hyperplasia (BPH), also referred to as benign prostatic enlargement (BPE), is a common condition in aging men that is associated with lower urinary tract symptoms characterized by voiding and storage dysfunction. BPH/BPE primarily results from increased cellular proliferation and smooth muscle contraction within the prostate. Multiple factors contribute to its development, including aging, imbalances in sex hormones (estrogen/testosterone), metabolic syndrome, and atherosclerosis, which affects the lower urinary tract^1^. Testosterone, the principal circulating androgen that promotes prostate growth, is mainly produced in the testes. Moreover, male sex hormones, particularly testosterone, are considered key risk factors for the development of BPH/BPE^1^. Metabolic syndrome, a cluster of conditions comprising obesity, hyperlipidemia, hyperglycemia, and hypertension, is a well-established contributor to metabolic dysfunction^2^.

Hypertension is highly prevalent among older adults and exerts detrimental effects on several organs, including the heart, brain, kidneys, prostate, and testes^3–6^. Both hypertension and sympathetic overactivity have been implicated in the pathogenesis of BPH/BPE. Hypertension-related mechanisms, such as ischemia, oxidative stress, inflammation, and the overexpression of growth factors in the prostate, are believed to contribute to prostatic hyperplasia^6,7^. However, the combined effect of aging and hypertension on prostatic morphology and function remains insufficiently characterized. Spontaneously hypertensive rats (SHRs) serve as a well-established model of genetic hypertension. These rats exhibit prostatic ischemia and morphological abnormalities in the ventral prostate as early as 15 weeks of age^8–10^. Aging further induces hyperplastic and structural alterations in the ventral prostate of SHRs^10^. Compared with normotensive Wistar Kyoto rats (WKYs), SHRs demonstrate a higher proliferation rate of epithelial cells in the ventral prostate^11^. However, studies addressing age-related variations in prostate weight and morphology in SHRs remain limited. This study, therefore, aimed to elucidate the effects of aging on the prostates and testes of SHRs, with emphasis on morphological, hemodynamic, and hormonal parameters of these changes.

## Results

### Prostate parameters

**Fig. 1:**
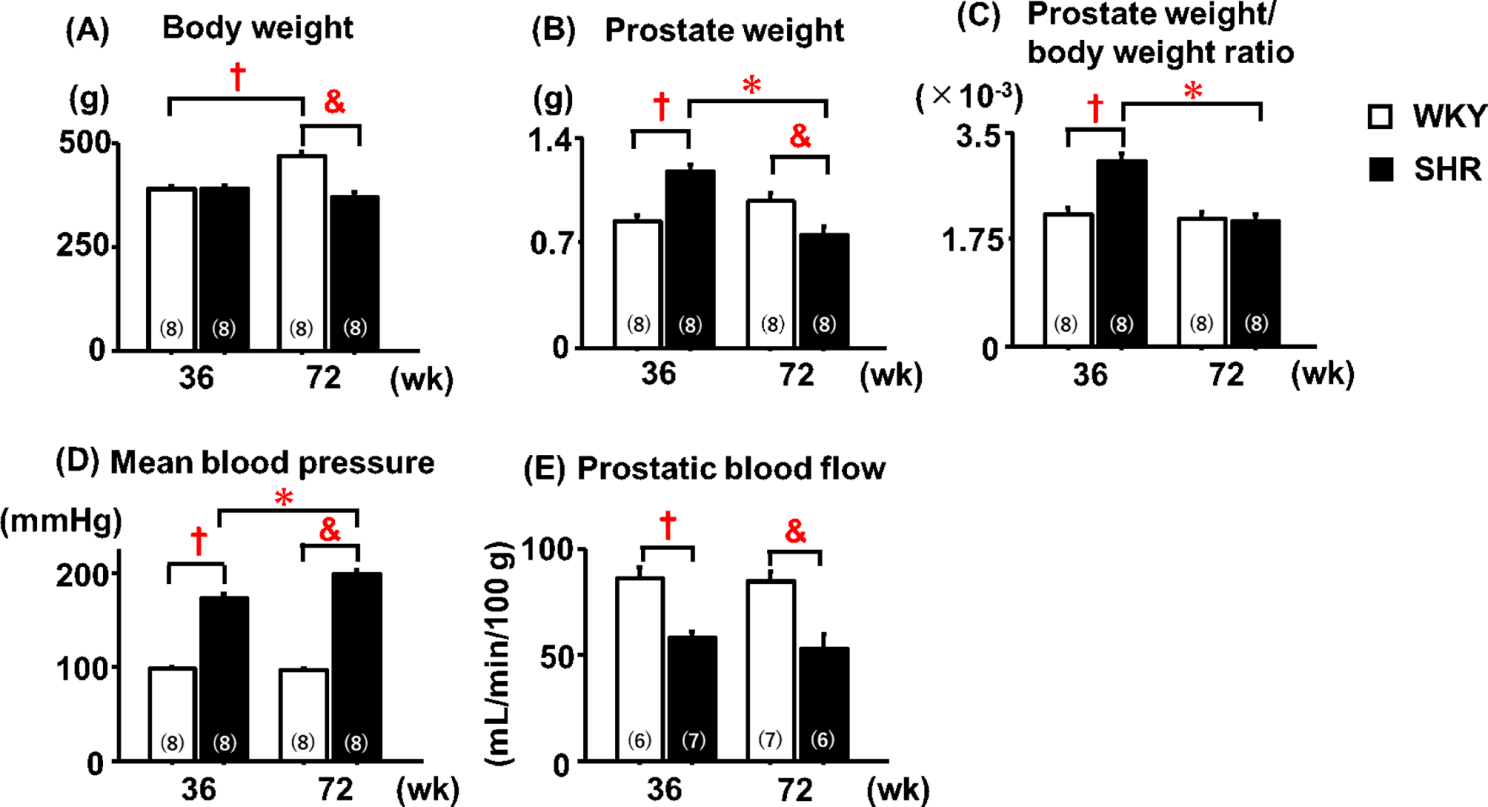
General parameters of the prostate. †: 36-week-old WKY; *: 36-week-old SHR; &: 72-week-old WKY. Data are expressed as the mean ± SEM (n = 6–8). Statistical significance shown in the figure was determined using the Tukey–Kramer post hoc test following two-way analysis of variance. Differences were considered statistically significant at *P* < 0.05.

There was no significant difference in body weight between adult WKYs and SHRs (36 weeks) (Fig. 1A). Compared with age-matched WKYs, adult SHRs exhibited significantly higher prostate weight and prostate weight-to-body weight ratio (Fig. 1B, C). In contrast, aged SHRs (72 weeks) demonstrated significantly lower body weight and prostate weight than age-matched WKYs (Fig. 1A–C). However, no significant difference in the prostate weight-to-body weight ratio was observed between aged SHRs and aged WKYs (Fig. 1C). Within the SHR group, aged SHRs displayed significantly lower prostate weight and prostate weight-to-body weight ratio than adult SHRs (Fig. 1B, C). At both ages, SHRs had significantly higher mean blood pressure and lower prostatic blood flow than age-matched WKYs, and an aging-dependent elevation of the mean blood pressure was observed in SHRs but not in WKYs (Fig. 1D, E). In contrast, prostate weight, prostate weight-to-body weight ratio, mean blood pressure, and prostatic blood flow did not differ significantly between adult and aged WKYs (Fig. 1B–E).

### Histology of the ventral prostate

**Fig. 2:**
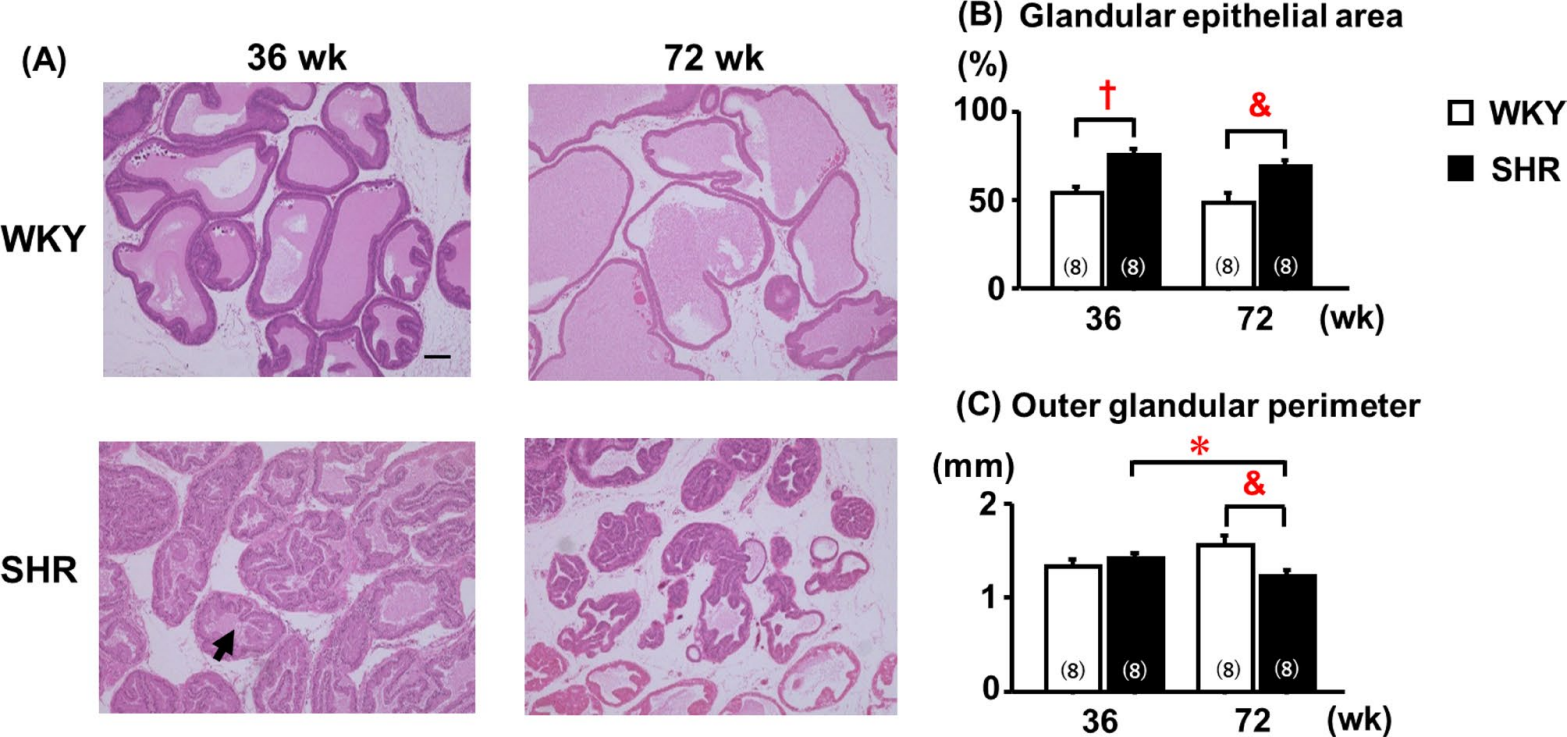
Age-related morphological changes in the ventral prostate. †: 36-week-old WKY; *: 36-week-old SHR; &: 72-week-old WKY. Quantitative data are expressed as the mean ± SEM (n = 8). The glandular epithelial area was evaluated as an indicator of prostatic hyperplasia, and the outer glandular perimeter was assessed as a marker of glandular atrophy. Histological scores were analyzed using the nonparametric Mann–Whitney U test with Bonferroni correction for multiple comparisons. Representative histological images are shown at 100 × magnification. Scale bar = 100 μm. Black arrow indicates luminal area in the gland. Differences were considered statistically significant at *P* < 0.05.

Compared with age-matched WKYs, both adult and aged SHRs exhibited more pronounced glandular morphological abnormalities in the ventral prostate, characterized by increased epithelial branching, a larger glandular epithelial area and lower luminal area in the gland. However, the glandular epithelial area did not differ significantly between adult and aged WKYs (Fig. 2A, B). Aged SHRs demonstrated a significantly smaller outer glandular perimeter than their age-matched WKYs or adult SHRs (Fig. 2A–C).

### Serum testosterone and testicular weight

**Fig. 3:**
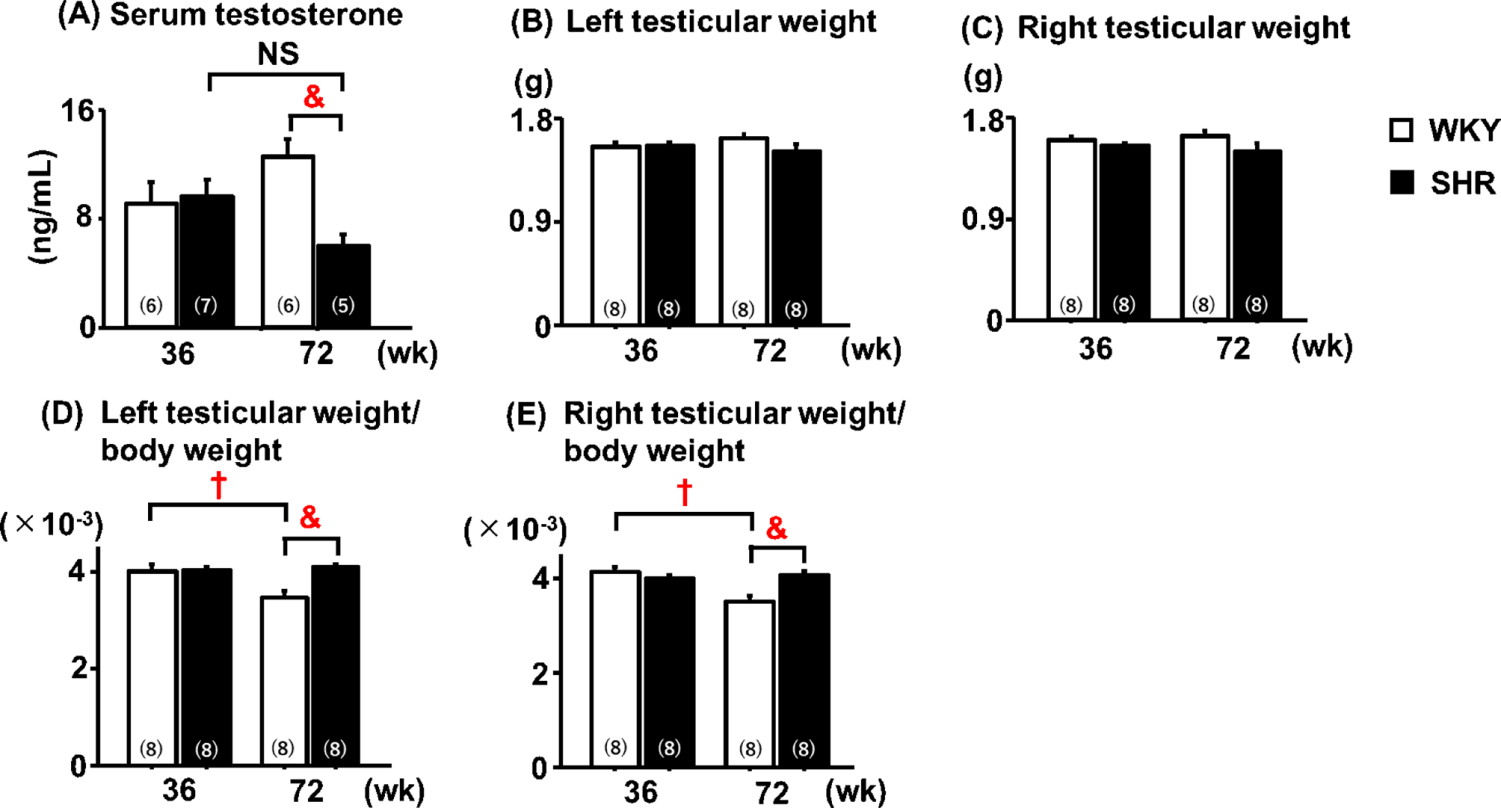
Serum testosterone levels and testicular parameters. †: 36-week-old WKY; &: 72-week-old WKY. NS: not significant. Data are expressed as the mean ± SEM (n = 5–8). Statistical significance shown in the figure was determined using the Tukey–Kramer post hoc test following two-way analysis of variance. Differences were considered statistically significant at *P* < 0.05.

Aged SHRs, but not adult SHRs, exhibited significantly lower serum testosterone levels than their age-matched WKYs (Fig. 3A). Although no significant difference in left or right testicular weight was observed between SHRs and WKYs at each age (Fig. 3B, C), aged SHRs showed significantly higher left and right testicular weight-to-body weight ratios than WKYs of the same age (Fig. 3D, E). In addition, no significant difference in the testicular weight-to-body weight ratio was observed between adult SHRs and adult WKYs (Fig. 3D, E).

### Histology of the testis

**Fig. 4:**
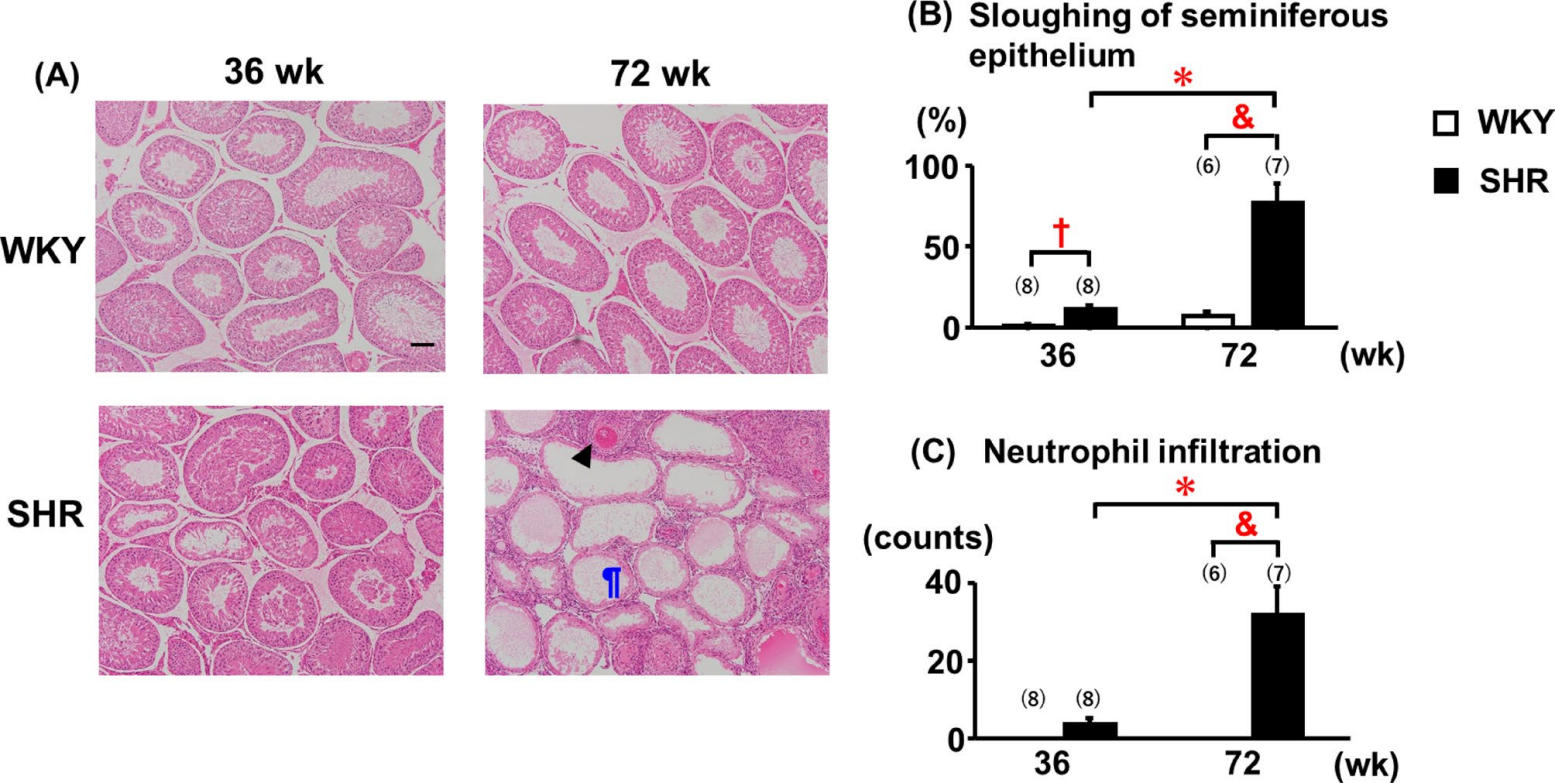
Age-related morphological changes in the testis. †: 36-week-old WKY; *: 36-week-old SHR; &: 72-week-old WKY. Data are expressed as the mean ± SEM (n = 6–8). Histological scores were analyzed using the nonparametric Mann–Whitney U test with Bonferroni correction for multiple comparisons. Representative histological images are shown at 100 × magnification. Scale bar = 100 μm. ¶: sloughing of the seminiferous epithelium; black arrowhead: thickening of blood vessels. Differences were considered statistically significant at *P* < 0.05.

Adult SHRs exhibited thickening of the testicular vascular wall, although the seminiferous tubules displayed minimal structural damage or germ cell loss compared with those of age-matched WKYs (Fig. 4A-C). In contrast, relative to both age-matched WKYs and adult SHRs, aged SHRs presented with more severe vascular wall thickening, marked sloughing of the seminiferous epithelium, and greater neutrophil infiltration (Fig. 4A–C).

## Discussion

Aging-related changes in prostate weight and morphology in SHRs have not yet been fully elucidated. In this study, adult SHRs exhibited higher blood pressure, an increased prostate weight-to-body weight ratio, and a larger glandular epithelial area compared with those in age-matched WKYs. In addition, aged SHRs demonstrated markedly higher blood pressure and greater glandular epithelial area but significantly lower prostate weight and outer glandular perimeter than WKYs of the same age. Although no significant difference in the prostate weight-to-body weight ratio was observed between aged SHRs and aged WKYs, reductions in prostate weight and glandular size suggest that aged SHRs develop more pronounced prostatic atrophy than their age-matched counterparts. Furthermore, aged SHRs exhibited higher blood pressure, lower prostate weight, and prostate weight-to-body weight ratios, and lower outer glandular perimeter than adult SHRs, indicating that aging is associated with the progression of severe hypertension in SHRs. Collectively, these findings suggest that adult SHRs develop glandular epithelial hyperplasia, whereas aged SHRs exhibit a transition from hyperplasia to atrophy of the prostate gland. Importantly, aging-related severe hypertension may contribute to the development of prostatic atrophy.

To our knowledge, this study is the first to evaluate age-associated prostatic morphological alterations in SHRs older than 54 weeks. A previous investigation reported that, compared with SHRs aged 15 weeks, those aged 54 weeks displayed a higher percent area density of epithelial and stromal components in the ventral prostate^10^. In our previous study, the survival rate of SHRs was 100% at 54 weeks but declined to 46.7% at 72 weeks, whereas WKYs maintained a 100% survival rate at 72 weeks^12^. Therefore, we selected 72-week-old rats to comprise the “aged” group, which represents an advanced stage of aging while maintaining adequate animal numbers for reliable analysis. In contrast, 36-week-old rats were classified as the “adult” group in this study.

In humans, BPH typically develops during midlife, whereas prostatic atrophy becomes common in older men. Therefore, the findings in SHRs may parallel these clinical patterns. Prostatic atrophy is frequently observed in older men^13,14^. Histologically, it is characterized by crowded glandular units with reduced cytoplasmic and nuclear volume compared with normal prostate tissue^15^. Although the precise mechanisms remain incompletely understood, prostatic atrophy has been associated with aging, antiandrogen therapy, inflammation, radiation exposure, and chronic ischemia resulting from local atherosclerosis^15,16^. Testosterone, the principal androgen synthesized in the testes, is essential for maintaining prostatic structure and function^17,18^. Androgen-deprivation therapy for prostate cancer reduces circulating testosterone concentrations to suppress tumor growth^17^. Similarly, pharmacologic treatments for BPH, such as 5α-reductase inhibitors (e.g., finasteride), lower dihydrotestosterone levels and cause prostate shrinkage^19–21^.

A previous study reported nearly 90% lower serum testosterone levels in aged SHRs (18–22 months) compared with young SHRs (16 weeks)^22^. Consistent with these findings, our study demonstrated significantly lower serum testosterone levels in aged SHRs than in age-matched normotensive WKYs. Because testosterone is primarily synthesized in the testes, hypertension can compromise male reproductive organs, including testicular function. However, the role of aging in hypertension-related testicular injury remains uncertain. Previous research has shown that SHRs exhibit both genetic hypertension and testicular pathology^23–25^. In the present study, both adult and aged SHRs displayed more testicular alterations, including vascular thickening and neutrophil infiltration, compared to those in age-matched WKYs, with greater severity observed in aged SHRs. These findings suggest that aging aggravates hypertension-induced testicular injury and reduces serum testosterone concentrations. Consequently, aging and hypertension may together contribute to reduced prostate weight and diminished gland size through testicular injury and androgen depletion.

Aging-related severe hypertension may be a key determinant in the development of prostatic atrophy. Consistent with our findings, a recent study reported that pulmonary arterial hypertension induces prostatic atrophy in rats^26^. Furthermore, similar outcomes have been documented in diabetic animal models. For instance, rats with streptozotocin-induced type 1 diabetes exhibit reduced prostate weight and lower serum testosterone levels^27–30^. Diabetes-induced hypoinsulinemia leads to marked reductions in prostate size. Structural remodeling of the stromal compartment in the ventral prostate has also been reported in alloxan-induced diabetic rats, with partial restoration following insulin replacement^31^. In these models, decreased testosterone concentrations are closely correlated with reduced prostate weight. Interestingly, the prostatic phenotype observed in diabetic rats resembles that seen in hypertensive models, suggesting shared mechanisms between androgen deficiency and organ atrophy. In humans, an association between hypertension and prostatic atrophy has not been clearly reported. However, current study suggests that aging-related severe hypertension may potentially contribute to prostatic atrophy through indirect mechanisms, such as declines in serum testosterone levels.

One limitation of the present study is that the underlying molecular mechanisms responsible for the reduction in prostate weight and the associated histological atrophic changes were not comprehensively investigated. While histological and morphometric analyses provided consistent and quantitative evidence of structural alterations, additional molecular studies will be required to elucidate the cellular and signaling mechanisms involved. Nevertheless, to our knowledge, this is the first study to demonstrate that aging in SHRs results not only in glandular epithelial hyperplasia but also in subsequent prostatic atrophy. Although a decline in serum testosterone is likely a major contributing factor, further studies will be necessary to clarify the downstream pathways contributing to these age-related pathological changes. Furthermore, whether pharmacological blood pressure control can mitigate prostatic atrophy and testicular impairment remains an important topic for future investigation.

## Conclusion

Aging and hypertension may jointly contribute to prostatic atrophy and decreased prostate weight through testicular injury and subsequent androgen depletion. Aging-related severe hypertension appears to be an important risk factor for prostatic atrophy, potentially accelerating the transition from hyperplasia to atrophy (Supplementary fig. S1). These findings offer valuable insight into the shared pathophysiological mechanisms underlying BPH and prostatic atrophy in humans.

## Methods

### Ethical approval

All animal procedures were approved by the Animal Care and Use Committee and the President of Kochi University (Approval Nos. I-2, J-32, K-17, L-14, N-13, O-14, and P-3) and were conducted in accordance with the ARRIVE guidelines and the National Institutes of Health standards^32^.

### Animals and housing

Male SHRs and WKYs (6–8 weeks old; Japan SLC Inc., Hamamatsu, Japan) were housed at a controlled temperature of 21–25 °C and relative humidity of 30–80% with a 14-h light/10-h dark cycle. Food (CE-2, CLEA Japan) and water were provided ad libitum until experimentation. All procedures complied with institutional welfare policies to minimize animal distress and reduce the number of animals used. The animals in this study were derived from the same cohort as a previous investigation that examined bladder function^12^. In this study, prostatic and testicular morphology and function were analyzed at distinct physiological endpoints. For ethical consistency and reduction of redundant animal use, baseline data (body weight and blood pressure) were obtained from the same animals as those used in the earlier report^12^. These data were reanalyzed to establish the context for the present investigation. No bladder-related data or figures were reused. Accordingly, this study represents an independent experiment with distinct objectives, analyses, and outcomes.

### Experimental procedure

Male SHRs were compared with age-matched WKY serving as normotensive controls at 36 and 72 weeks of age. The mean blood pressure was measured at each time point using the tail-cuff method^33^. Body weight was recorded, and rats were anesthetized with urethane (1.0 g/kg, intraperitoneally). Prostatic blood flow was assessed using the hydrogen-clearance method^33^. Blood samples were collected from the vena cava, after which the animals were euthanized via an overdose of sodium pentobarbital (200 mg/kg, intraperitoneally). The prostates and testes were excised and weighed. The different parts of tissue were fixed and processed for hematoxylin and eosin staining and histological evaluation.

### Histological evaluation of the ventral prostate

Ventral prostate tissues were fixed and stained with hematoxylin and eosin. The glandular epithelial area was quantified as the ratio of the stained epithelial area to the total glandular area, as described in our previous studies^33,34^. The glandular epithelial area was calculated by subtracting the luminal area from the total glandular area. The glandular epithelial area (%) was normalized to the total glandular area and expressed as a percentage using the following formula: glandular epithelial area (%) = (glandular area − luminal area) / glandular area × 100.

(Supplementary fig. S2). ImageJ software was used for quantitative analysis, and the glandular epithelial area served as an indicator of glandular epithelial hyperplasia^33,34^. The outer glandular perimeter in prostate was measured using a Keyence BZ-X800 image analysis system (Keyence, Japan) as an indicator of gland size (Supplementary fig. S3). Twenty glands were randomly selected per rat, and the mean value was used for statistical analysis.

### Measurement of serum testosterone

Serum testosterone concentrations were determined using an enzyme-linked immunosorbent assay kit (R&D Systems, KGE010, Minneapolis, USA). Optical density was measured at 450 nm within 30 min using a microplate reader.

### Histological evaluation of the testis

Left testis samples were fixed and stained with hematoxylin and eosin. Histological evaluation was performed by an experienced pathologist (A.K.) who was blinded to group allocation. Sloughing of the seminiferous epithelium was assessed in 100 tubules per sample. Neutrophil infiltration, predominantly observed around blood vessels with fibrinoid degeneration, was evaluated in five randomly selected high-power fields (400 × magnification), and the number of neutrophils per field was counted.

### Statistical analysis

Group sizes were determined based on expected effect sizes and standard errors derived from preliminary experiments and previous studies^33,34^. Data are expressed as the mean ± standard error of the mean. Statistical comparisons for non-histological parameters were performed using two-way analysis of variance (Supplementary table 1), followed by the Tukey–Kramer post hoc test. Histological data were analyzed using the nonparametric Mann–Whitney U test with Bonferroni correction for multiple comparisons. All statistical analyses were performed using Prism and StatView software. Statistical significance was defined as *P* < 0.05.

## Supplementary Information

Below is the link to the electronic supplementary material.

Supplementary Material 1

Supplementary Material 2

Supplementary Material 3

Supplementary Material 4

## Data Availability

The datasets generated or analyzed during the current study are available from the corresponding author upon reasonable request.
