# Supplementary material for: Aging-related severe hypertension causes prostatic gland atrophy and testicular injury in rats
Source: Sci Rep. 2026 Mar 3;16:11902. doi: 10.1038/s41598-026-41624-x (PMC13065736; doi:10.1038/s41598-026-41624-x)
Supplement: Supplementary file 1 — Supplementary Material 1 [file 41598_2026_41624_MOESM1_ESM.docx]

| **Outcome** | **Factor** | **Df** | **F value** | **P value** |
| --- | --- | --- | --- | --- |
| Body weight | Age | 1,28 | 8.83 | 0.006 |
|  | Hypertension | 1,28 | 27.13 | <0.001 |
|  | Interaction | 1,28 | 25.82 | <0.001 |

**Table 1. Two-way ANOVA results for body weight**

For body weight, two-way ANOVA revealed significant main effects of age and hypertension, as well as a significant interaction between these factors. Df: degree of freedom.

**Table 2. Two-way ANOVA results for prostate weight**

| **Outcome** | **Factor** | **Df** | **F value** | **P value** |
| --- | --- | --- | --- | --- |
| Prostate weight | Age | 1,28 | 8.05 | 0.008 |
|  | Hypertension | 1,28 | 1.27 | 0.270 |
|  | Interaction | 1,28 | 29.57 | <0.001 |

For prostate weight, two-way ANOVA revealed significant main effect of age, as well as a significant interaction between age and hypertension. Df: degree of freedom.

**Table 3.** **Two-way ANOVA results for prostate weight/body weight ratio**

| **Outcome** | **Factor** | **Df** | **F value** | **P value** |
| --- | --- | --- | --- | --- |
| Prostate weight/body weight ratio | Age | 1,28 | 21.88 | <0.001 |
|  | Hypertension | 1,28 | 12.7 | 0.001 |
|  | Interaction | 1,28 | 15.23 | <0.001 |

For prostate weight/body weight ratio, two-way ANOVA revealed significant main effects of age and hypertension, as well as a significant interaction between these factors. Df: degree of freedom.

**Table 4.** **Two-way ANOVA results for mean blood pressure**

| **Outcome** | **Factor** | **Df** | **F value** | **P value** |
| --- | --- | --- | --- | --- |
| Mean blood pressure | Age | 1,28 | 8.43 | 0.007 |
|  | Hypertension | 1,28 | 507.6 | <0.001 |
|  | Interaction | 1,28 | 10.41 | 0.003 |

For mean blood pressure, two-way ANOVA revealed significant main effects of age and hypertension, as well as significant interaction between these factors. Df: degree of freedom.

**Table 5.** **Two-way ANOVA results for prostatic blood flow**

| **Outcome** | **Factor** | **Df** | **F value** | **P value** |
| --- | --- | --- | --- | --- |
| Prostatic blood flow | Age | 1,22 | 0.62 | 0.439 |
|  | Hypertension | 1,22 | 31.79 | <0.001 |
|  | Interaction | 1,22 | 0.22 | 0.644 |

For prostatic blood flow, two-way ANOVA revealed a significant main effect of hypertension, whereas neither the main effect of age nor the interaction between age and hypertension was significant. Df: degree of freedom.

**Table 6.** **Two-way ANOVA results for serum testosterone**

| **Outcome** | **Factor** | **Df** | **F value** | **P value** |
| --- | --- | --- | --- | --- |
| Serum testosterone | Age | 1,20 | 0.01 | 0.927 |
|  | Hypertension | 1,20 | 5.22 | 0.033 |
|  | Interaction | 1,20 | 7.13 | 0.015 |

For serum testosterone, two-way ANOVA revealed significant main effects of hypertension, as well as a significant interaction between age and hypertension. Df: degree of freedom.

**Table 7.** **Two-way ANOVA results for left testicular weight**

| **Outcome** | **Factor** | **Df** | **F value** | **P value** |
| --- | --- | --- | --- | --- |
| Left testicular weight | Age | 1,28 | 0.09 | 0.772 |
|  | Hypertension | 1,28 | 1.33 | 0.259 |
|  | Interaction | 1,28 | 2.12 | 0.157 |

For left testicular weight, two-way ANOVA revealed no significant main effects of age or hypertension, nor a significant interaction between age and hypertension. Df: degree of freedom.

**Table 8.** **Two-way ANOVA results for right testicular weight**

| **Outcome** | **Factor** | **Df** | **F value** | **P value** |
| --- | --- | --- | --- | --- |
| Right testicular weight | Age | 1,28 | 0.05 | 0.822 |
|  | Hypertension | 1,28 | 3.52 | 0.071 |
|  | Interaction | 1,28 | 0.65 | 0.429 |

For right testicular weight, two-way ANOVA revealed no significant main effects of age or hypertension, nor a significant interaction between age and hypertension. Df: degree of freedom.

**Table 9.** **Two-way ANOVA results for left testicular weight/body weight ratio**

| **Outcome** | **Factor** | **Df** | **F value** | **P value** |
| --- | --- | --- | --- | --- |
| Left testicular weight/body weight ratio | Age | 1,28 | 3.72 | 0.064 |
|  | Hypertension | 1,28 | 7.92 | 0.009 |
|  | Interaction | 1,28 | 6.25 | 0.019 |

For left testicular weight/body weight ratio, two-way ANOVA revealed significant main effect of hypertension, as well as a significant interaction between age and hypertension. Df: degree of freedom.

**Table 10.** **Two-way ANOVA results for right testicular weight/body weight ratio**

| **Outcome** | **Factor** | **Df** | **F value** | **P value** |
| --- | --- | --- | --- | --- |
| Right testicular weight/body weight ratio | Age | 1,28 | 6.73 | 0.015 |
|  | Hypertension | 1,28 | 3.84 | 0.060 |
|  | Interaction | 1,28 | 9.89 | 0.004 |

For right testicular weight/body weight ratio, two-way ANOVA revealed significant main effect of age, as well as a significant interaction between age and hypertension. Df: degree of freedom.
