# Supplementary figures and images for: Aging-related severe hypertension causes prostatic gland atrophy and testicular injury in rats
Source: Sci Rep. 2026 Mar 3;16:11902. doi: 10.1038/s41598-026-41624-x (PMC13065736; doi:10.1038/s41598-026-41624-x)

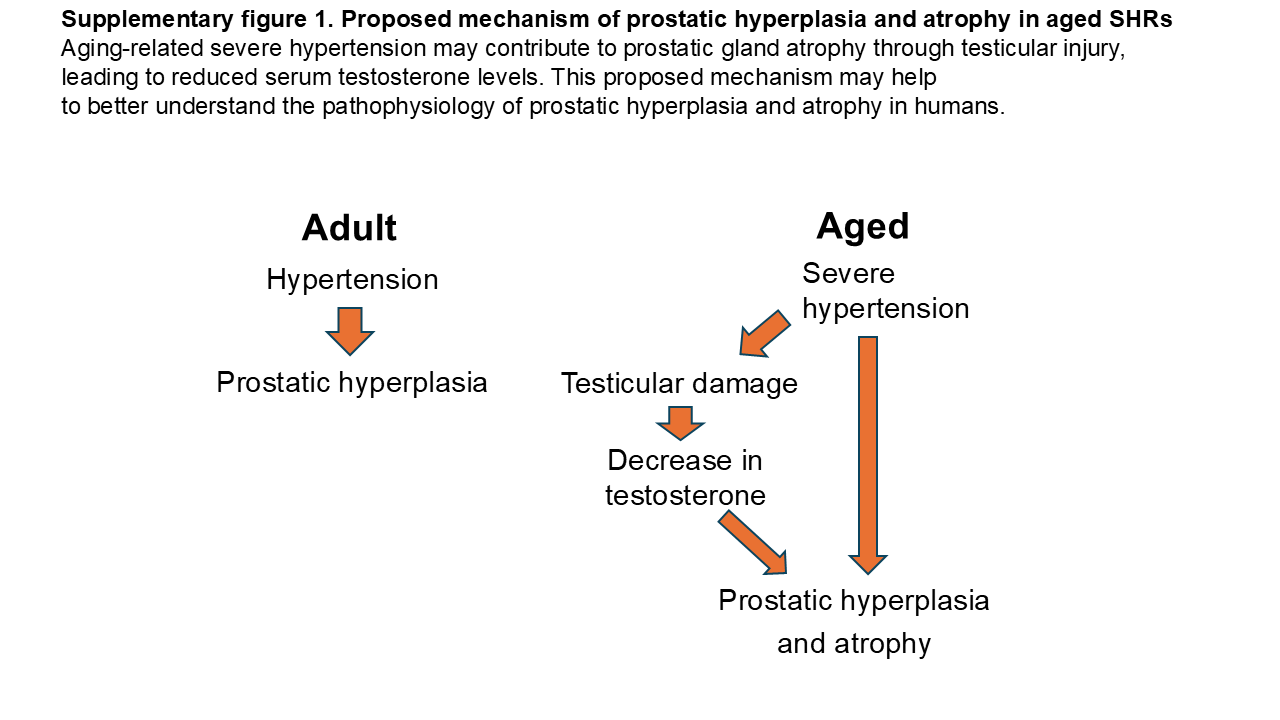

Supplement: Supplementary file 2 — Supplementary Material 2 [file 41598_2026_41624_MOESM2_ESM.tif]

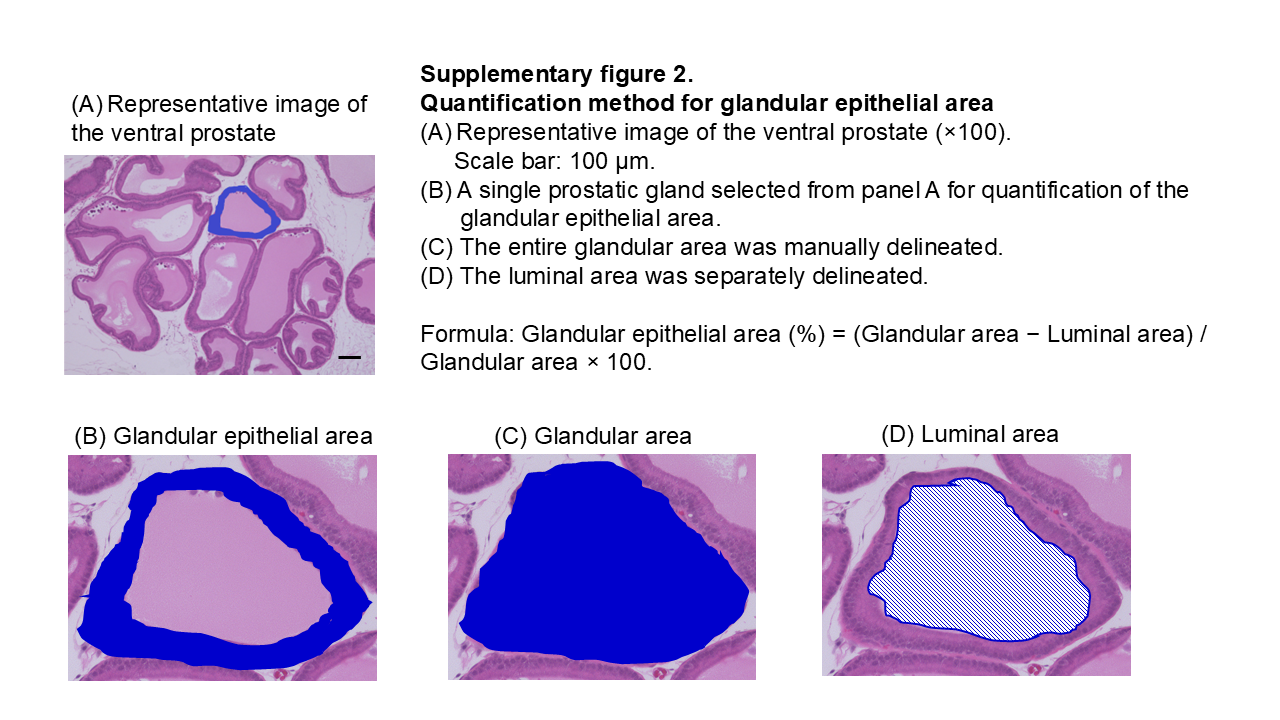

Supplement: Supplementary file 3 — Supplementary Material 3 [file 41598_2026_41624_MOESM3_ESM.tif]

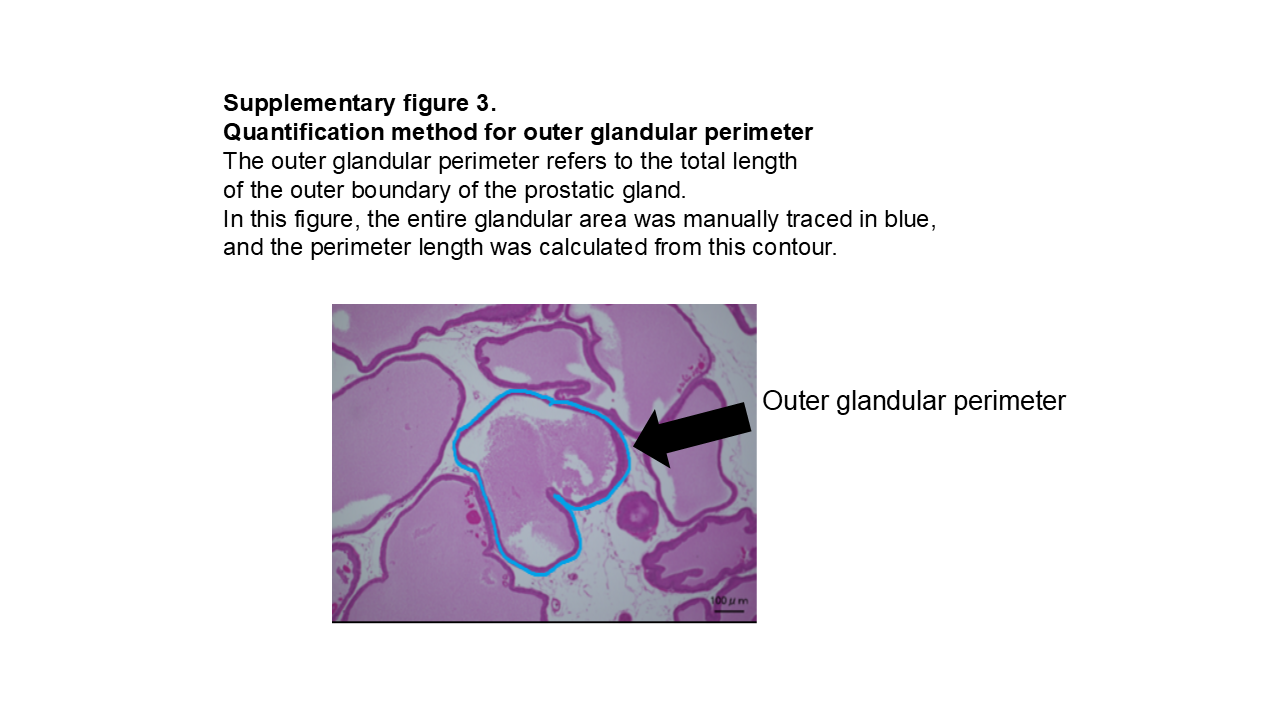

Supplement: Supplementary file 4 — Supplementary Material 4 [file 41598_2026_41624_MOESM4_ESM.tif]
